# Economic Sanctions on Iran and Nuclear Medicine

**DOI:** 10.22038/AOJNMB.2018.36919.1248

**Published:** 2019

**Authors:** S. Rasoul Zakavi

**Affiliations:** Nuclear Medicine Research Center, Mashhad University of edical Sciences, Mashhad, Iran

“It is not a wise choice!”, this was the reaction of my father when I applied for nuclear medicine residency program 26 years ago. The old retired officer continued that hi-tech nuclear medicine is dependent on multiple advanced sections that may not be easily available especially in the developing countries. Now he is not alive to see that political misconducts have added fuel to the fire.

Global shortage of Technetium-99m in recent years revealed the vulnerability of nuclear medicine and dependency of our clinical departments on the policies of the governments to support production of radiotracers (1). Although the mission of International Atomic Energy Agency (IAEA) is to “accelerate and enlarge the contribution of atomic energy to peace, health, and prosperity throughout the world”, its application is highly affected by local governmental policies (2). 

Recent unilateral withdrawal of USA from Iran nuclear deal (Joint Comprehensive Plan Of Action-JCPOA) followed by imposing economic, trade and financial sanctions against Iran, has deleterious effect on nuclear medicine either on supply of radiotracers or spare parts of nuclear medicine devices (3). Although medicine is apparently not included in the list of sanctions, secondary sanction, aviation and transport embargo as well as financial restrictions, made it extremely difficult for medical companies to be able to do any transaction. Payment for the drugs or instruments and shipment of the goods to and from Iran have turned to a lengthy, difficult and risky task. Nuclear medicine seems to be at particular risk due to its link with atomic energy agency.

Multiple reports in the literature have been indicated the harmful effect of previous economic sanctions, on health of Iranian patients (4-9). It has strangled Iranian’s health, disrupted drug supply and negatively affected millions of patients(10). It has shown that the negative effect of sanctions has ranged from death to different complications of disease mainly due to limited access to the drugs. The most critical patients have been affected the worst including children, patients with cancer, hemophilia, cardiovascular disease, asthma and epilepsy (11-14). Nuclear medicine plays important role in diagnosis and treatment of these patients. It seems that among different disciplines of medicine, nuclear medicine is the weakest link in critical conditions. Supply of radiopharmaceuticals, imaging instruments and their spare parts disrupted in previous sanction in 2012 and nuclear medicine departments in Iran were shut down for about 45 days before being able to secure a new source for Molybdenum(15).

Furthermore, economic sanctions have been resulted in remarkable inflation that kept most of hi-tech diagnostic and therapeutic modalities out of reach of patients with poor economic conditions. Not to mention that drastic devaluation of the national currency, moved large number of people from middle to lower economic classes and they could not afford medical costs anymore (16).

Iranian experiences from previous sanctions however urged them for local production of radiopharmaceuticals and assemble of radioactive generators. Currently a wide variety of radiopharmaceuticals are locally produced including nearly all types of cold kits, ^131^I, ^201^Tl-chloride, ^67^Ga-citrate, ^153^Sm-EDTMP, ^177^Lu-EDTMP, ^177^Lu-Octreotate, ^177^Lu-PSMA,^ 90^Y-citrate, ^90^Y-hydroxyapatite colloid, ^188/186^Re-sulfur colloid, and ^188/186^Re-HEDP as well as Mo-99/Tc-99m Generators, Ge-68/Ga-68 generators and Rb-81/Kr-81m (17). Due to competitive prices of these products, many nuclear medicine departments in developing countries have been importing these radiopharmaceuticals from Iran. Currently, Iran is one of the suppliers of radiopharmaceuticals in Iraq, India, Pakistan, Syria, Egypt, Georgia and few other Asian, and European countries(18). However raw materials have been imported for these productions and secure air transport is mandatory to export the final products, so any sanction in this sections will hamper supply of radiopharmaceuticals in the region far beyond Iran.

Economic sanctions has been used as a tool of pressure in political disputes against many countries including Iraq, Russia, Syria, Iran, Cuba, Venezuela, North Korea and Haiti and have not resulted to regime change in any of these countries. However there is clear evidence that they resulted in death and severe health problems for many patients in these countries (19-21). As people has the basic right to health, these sanctions can be considered as a violation of basic human rights (4, 22). Human suffering must not be used as a tool for political achievements(22).

In conclusion, recent USA economic sanctions on Iran have been adversely affected practice of nuclear medicine and patient care in the country, that might extend beyond national borders.
